# Development and Implementation of an Antimicrobial Stewardship Checklist in Sub-Saharan Africa: A Co-Creation Consensus Approach

**DOI:** 10.3390/healthcare10091706

**Published:** 2022-09-06

**Authors:** Diane Ashiru-Oredope, Frances Garraghan, Omotayo Olaoye, Eva M. Krockow, Ayodeji Matuluko, Winnie Nambatya, Peter Ahabwe Babigumira, Chloe Tuck, George Amofah, Daniel Ankrah, Scott Barrett, Peter Benedict, Kwame Peprah Boaitey, Kwame Ohene Buabeng, Sarah Cavanagh, Esmita Charani, Enock Chikatula, Sam Ghebrehewet, Jasmin Islam, Yogini H. Jani, Esther Johnston, Mohammed Lamorde, Augustine Malinga, Mariyam Mirfenderesky, Victoria Rutter, Jacqueline Sneddon, Richard Skone-James

**Affiliations:** 1 Commonwealth Pharmacists Association, London E1W 1AW, UK; 2 HCAI and AMR Division, UK Health Security Agency, Wellington House, London SW1 8UG, UK; 3 Pharmacy Department, Manchester University NHS Foundation Trust, Oxford Road, Manchester M13 9WL, UK; 4 Department of Neuroscience, Psychology & Behaviour, University of Leicester, University Road, Leicester LE1 7RH, UK; 5 Department of Pharmacy, Makerere University, Wandegeya, Makerere, Kampala P.O. Box 7062, Uganda; 6 The Infectious Diseases Institute, Kampala P.O. Box 22418, Uganda; 7 Ghana Public Health Association, Ghana Public Health Association Liberia Link, Accra GA-107-5253, Ghana; 8 Korle-Bu Teaching Hospital, Accra P.O. Box 77, Ghana; 9 Pharmacy Department, North Tyneside Hospital, Northumbria Healthcare NHS Foundation Trust, Rake Lane, North Shields NE29 8NH, UK; 10 Pharmacy Department, Kilimanjaro Christian Medical Centre (KCMC), Moshi P.O. Box 3010, Tanzania; 11 Institute for Evidence-Based Healthcare, Bond University, Gold Coast, QLD 4226, Australia; 12 Department of Pharmacy Practice, Faculty of Pharmacy and Pharmaceutical Sciences, College of Health Sciences, Kwame Nkrumah University of Science and Technology, Kumasi UPO, Ghana; 13 Faculty of Medicine, Department of Infectious Disease, Imperial College London, London SW7 2AZ, UK; 14 Pharmacy Department, University Teaching Hospital (UTH), Lusaka Private Bag RW1X, Zambia; 15 North West Region, UK Health Security Agency, Wellington House, 133-155 Waterloo Road, London SE1 8UG, UK; 16 Brighton and Sussex Medical School (BSMS), University of Sussex, Brighton BN1 9PX, UK; 17 Centre for Medicines Optimisation Research and Education, University College London Hospitals NHS Foundation Trust, 250 Euston Road, London NW1 2PG, UK; 18 Norfolk and Suffolk NHS Foundation Trust, The Gate Lodge, 177 Ribbans Park Road, Ipswich IP3 8XL, UK; 19 North Middlesex University Hospital NHS Trust, London N18 1QX, UK; 20 Scottish Antimicrobial Prescribing Group, Healthcare Improvement Scotland, Delta House, 50 West Nile Street, Glasgow G1 2NP, UK; 21 Tropical Health and Education Trust, 1 St. Andrews Place, Regent’s Park, London NW1 4LE, UK

**Keywords:** AMS checklist, antimicrobial prescribing, CwPAMS, Global-PPS, antimicrobial stewardship

## Abstract

Antimicrobial stewardship (AMS) initiatives promote the responsible use of antimicrobials in healthcare settings as a key measure to curb the global threat of antimicrobial resistance (AMR). Defining the core elements of AMS is essential for developing and evaluating comprehensive AMS programmes. This project used co-creation and Delphi consensus procedures to adapt and extend the existing published international AMS checklist. The overall objective was to arrive at a contextualised checklist of core AMS elements and key behaviours for use within healthcare settings in Sub-Saharan Africa, as well as to implement the checklist in health institutions in four African countries. The AMS checklist tool was developed using a modified Delphi approach to achieve local expert consensus on the items to be included on the checklist. Fourteen healthcare/public health professionals from Tanzania, Zambia, Uganda, Ghana and the UK were invited to review, score and comment on items from a published global AMS checklist. Following their feedback, 8 items were rephrased, and 25 new items were added to the checklist. The final AMS checklist tool was deployed across 19 healthcare sites and used to assess AMS programmes before and after an AMS intervention in 14 of the 19 sites. The final tool comprised 54 items. Across the 14 sites, the completed checklists consistently showed improvements for all the AMS components following the intervention. The greatest improvements observed were the presence of formal multidisciplinary AMS structures (79%) and the execution of a point-prevalence survey (72%). The elements with the least improvement were access to laboratory/imaging services (7%) and the presence of adequate financial support for AMS (14%). In addition to capturing the quantitative and qualitative changes associated with the AMS intervention, project evaluation suggested that administering the AMS checklist made unique contributions to ongoing AMS activities. Furthermore, 29 additional AMS activities were reported as a direct result of the prompting checklist questions. Contextualised, co-created AMS tools are necessary for managing antimicrobial use across healthcare settings and increasing local AMS ownership and commitment. This study led to the development of a new AMS checklist, which proved successful in capturing AMS improvements in Tanzania, Zambia, Uganda, and Ghana. The tool also made unique contributions to furthering local AMS efforts. This study extends the existing AMS materials for low- and middle-income countries and provides empirical evidence for successful use in practice.

## 1. Introduction

Antimicrobial stewardship (AMS) has been recommended as a key strategy for optimising the use of antimicrobials and reducing the global threat of antimicrobial resistance (AMR) [1]. AMS programmes have evolved in different healthcare settings [2,3,4], but most relevant research has been conducted in high-income countries, whose healthcare systems are supported by a political commitment to AMS and substantial financial investments [4]. Learning from high-income countries needs to be shared and adapted for low- and middle-income countries (LMICs). The initial successes provide evidence for the effectiveness of shared learning approaches [1,5,6,7,8,9,10]. A key requirement for the success of shared learning appears to be the engagement and empowerment of frontline staff, who need to be equipped with the skills and tools to carry out AMS effectively.

In 2019, Pulcini et al. [11] developed a global checklist of the core elements of hospital AMS programmes. The checklist collates information from published literature, the previously developed core elements of AMS programmes, and their accompanying checklist items. However, the authors themselves identified a number of shortcomings of their tool, for example, stating ‘… most of these checklist items may not currently exist in most hospitals in low-income countries’ and suggesting ‘These seven core elements and their related 29 checklist items could be adapted and adopted locally depending on factors such as clinical setting and resource availability’ [11] p. 23. Subsequent efforts have addressed these suggestions and focused on developing more appropriate materials for the LMIC context [12]. In October 2019, the WHO published a toolkit of essential national core elements for AMS programmes in LMICs. This was supplemented by a 28-item checklist of essential healthcare facility core elements for AMS programmes in LMICs, differentiating between the ‘basic’ and ‘advanced’ elements [12]. The development of materials for LMIC healthcare settings was an initial step toward more contextualised AMS approaches.

This study takes another step toward increasing the suitability and acceptance of standardised AMS tools in LMIC settings in several different ways:

(1) Our focus on Sub-Saharan Africa (specifically Tanzania, Zambia, Uganda, and Ghana) offers a further local adaptation of the materials.
(2) Our unique methodological approach uses elements of co-creation through the strong involvement of local hospital representatives during a Delphi consensus procedure.
(3) We extended the number of checklist items to capture the more nuanced differences in the AMS elements. Additionally, we incorporate open-ended questions within the AMS checklist to allow for more reporting flexibility.
(4) We provide an initial evaluation of the checklist’s effectiveness by using it to measure the outcomes of an AMS intervention programme.

The checklist was developed as part of the ‘Commonwealth Partnerships for Antimicrobial Stewardship’ (CwPAMS) programme, funded by UK aid Fleming Fund and jointly managed through the Tropical Health and Education Trust (THET) and the Commonwealth Pharmacists Association (CPA) [13,14,15,16]. The CwPAMS programme ran from inception in September 2018 until June 2021 and was set up to support 12 health partnerships between teams of volunteers (including pharmacists and specialist nurses) from the UK’s National Health Service (NHS) Trusts and higher education institutes and health workers in four African countries (Tanzania, Zambia, Uganda, and Ghana). The CwPAMS programme provided the perfect setting for developing the AMS checklist because the existing project infrastructure enabled the easy identification of representative healthcare workers to be included in the consensus process. Given CwPAMS’ efforts in running AMS interventions, the project further allowed us to test the success of the newly developed checklist.

## 2. Materials and Methods

### 2.1. Checklist Development

Pulcini et al.’s [11] global AMS checklist served as the baseline document for our project. The WHO’s AMS toolkit for LMICs had not been published at that time. Using Pulcini et al.’s original items as a starting point, we adopted a modified Delphi procedure for achieving a consensus on the items to be included in our contextualised AMS checklist for the Sub-Saharan healthcare context. The consensus procedure involved rating the importance of items as well as making open-ended comments and suggestions. The CwPAMS project structure was used to engage local hospital representatives across four countries in Sub-Saharan Africa.

**Figure 1:**
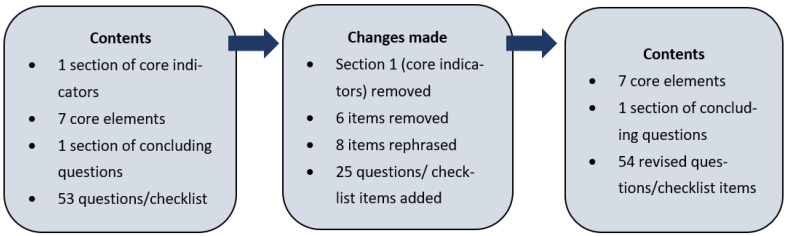
Summary of consensus process.

Fourteen healthcare representatives from the CwPAMS partnerships were invited from April 2019 to participate in the consensus process following the project inception training sessions with all the UK and African partnership leads involved in the CwPAMS project. The 14 representatives included eight CwPAMS health partnership leads, including pharmacists, public health specialists, and microbiologists from the UK and the four African countries, four healthcare professionals working in hospitals, national pharmacy and/or public health associations based in Ghana, and two healthcare professionals based in Uganda (working in a regional referral hospital and national research institute). The consensus process is summarised in Figure 1. Further details on the consensus process and the development of the AMS checklist are included in Supplementary Materials S1 to S3.

### 2.2. AMS Checklist Implementation across 19 Hospitals

Following a pilot with two partnership sites, the final AMS checklist was deployed as an online form in April 2019 across 19 sites in Sub-Saharan Africa, which included 14 CwPAMS project sites (6 Ghana, 6 Uganda, 1 Tanzania and 1 Zambia) and 5 regional referral centres in Uganda. Each hospital site provided information on the current state of their AMS activities based on the questions on the checklist. For the CwPAMS sites, the checklist was jointly completed through discussions between the respective UK and African lead partners. To facilitate this, a PDF or spreadsheet version of the checklist was made available. For the additional sites in Uganda, the pharmacists at each institution completed the checklist with support from independent colleagues with AMS expertise to discuss and complete the form with relevant individuals. The lead African and UK partners for each CwPAMS site completed the checklist again to provide updated information on the state of their AMS activities post-CwPAMS intervention. The respondents were also asked to include information on the members of their multidisciplinary AMS teams pre- and post-CwPAMS intervention.

### 2.3. Demographics of Study Sites

Across the 19 study sites, the hospitals averaged 536 inpatient beds, with the lowest number of hospital beds reported as 100 and the highest as 2000 inpatient beds (both in Ghana). Eight out of nineteen hospitals (42%) were tertiary hospitals, five (26%) were secondary hospitals, and five (26%) were regional referral hospitals. Only one site (5%) was a primary care institution. Twelve out of nineteen sites (63%) were teaching hospitals. The names of all participating hospital sites can be found in Supplementary Material S3.

## 3. Results

The final AMS checklist contained 54 items across eight main sections (Supplementary Material S2). These included seven sections on the core elements of hospital AMS programmes (senior management and leadership towards AMS; accountability and responsibilities; available expertise on infection management and stewardship; education and practical training; continual monitoring and surveillance; regular reporting and feedback; other actions aiming at responsible antimicrobial use) and the concluding section. It differed from the original checklist by Pulcini et al. [11] in several important ways.

The new items added: 24 new items were added to Pulicini et al.’s [11] original checklist. Most items were added in the sub-sections on accountability and responsibilities, education and practical training, and other actions aiming at responsible antimicrobial use. The added items reflected a stronger focus on the details around the AMS team, a more detailed assessment of induction training for clinical staff, and questions around local prescribing and the Infection Prevention and Control (IPC) protocols. The new items also included different question formats compared to Pulcini et al.’s [11] binary choice questions. The examples were open-ended questions (e.g., ‘Please provide more details about the AMS leader, and how much time is available to dedicate to AMS activities etc.’). Numerical questions (e.g., ‘What was the total number of each clinical staff trained in the last year’) and multiple-choice questions (e.g., ‘How is the training delivered? (Select all that apply)’) were also asked.

The items removed: Eight items were removed from the original checklist. Almost all of these items were part of the section on other actions aiming at responsible antimicrobial use. Item removal was determined by their relevance to Sub-Saharan healthcare settings and the limited availability of resources. An example of a deleted item includes: ‘Does your hospital support the antimicrobial stewardship activities/strategy with adequate information technology services?’

The items rephrased: Eight original checklist items were rephrased to increase understanding and better reflect the local healthcare contexts. For example, the original question ‘Are clinicians, other than those part of the antimicrobial stewardship team (e.g., from the ICU, Internal Medicine and Surgery) involved in the antimicrobial stewardship committee?’ was extended. The new question was phrased: (i.e., ‘Are clinicians, nurses or pharmacists, other than those part of the AMS team …’) to specifically include a focus on nurses and pharmacists.

### 3.1. Checklist Implementation

#### Quantitative Improvements Following the AMS Intervention

Table 1 shows a comparison of the core checklist results pre and post the CwPAMS AMS strengthening intervention, delivered through the partnerships. The five additional sites in Uganda (without the CwPAMS project interventions) only completed the checklist once and are therefore excluded from this comparison. Their checklist results can be found in a separate table in Supplementary Material S4.

Improvements were reported across all core indicators of the AMS checklist. The largest improvements pertained to the core AMS checklist element on the multidisciplinary organisational structures responsible for AMS. Before the AMS intervention, only three healthcare sites reported having such a formal AMS structure. After the intervention, all 14 sites gave positive answers to this question, indicating a 79% increase. Other notable improvements were observed regarding the conduct of point-prevalence surveys for antimicrobial use and the availability of multidisciplinary AMS teams to support the implementation of the AMS strategy.

Smaller improvements were reported for the elements of access to laboratory or imaging services (7%) and the existence of a dedicated, sustainable, and sufficient AMS budget (14%), with the overall number of healthcare sites remaining low, even after the intervention. Lesser improvements were also observed for some items that ranked high prior to the intervention (e.g., the availability of published IPC protocols). This may be explained by a ceiling effect, whereby little further improvement could be obtained on those comparatively well-established items.

Table 2 shows a detailed breakdown of the number of members (by profession) that formed part of the multidisciplinary AMS teams pre- and post-intervention. Apart from one exception (intensive care (ITU) consultants), increases could be observed across all professional categories. The largest increase was reported in the involvement of nurses and pharmacists, with 21 new members of those professions joining the multidisciplinary AMS teams over the course of the intervention.

### 3.2. Development and Review of Guidelines/Policies

The checklist also captured the new guideline development that resulted locally as a result of the CwPAMS intervention: Eight projects reported developing new documents (guidelines/policies/posters, etc.) focused on either AMS or antibiotic prescribing as a result of CwPAMS. Four of these projects reported developing two or more new AMS documents. Three projects reported that they had revised or updated documents (guidelines/policies/posters, etc.) focused on either AMS or antibiotic prescribing as a result of CwPAMS. Five projects reported that they had developed new documents (guidelines/policies/posters, etc.) focused on IPC as a result of CwPAMS. Three of these projects developed two or more new IPC documents. Three projects reported that they had revised or updated documents (guidelines/policies/posters, etc.) focused on IPC as a result of CwPAMS.

### 3.3. Raising Awareness of WHO AWaRe Categories

The AMS checklist reports indicated that 79% (11 out of 14) of the projects had increased awareness of the WHO’s AWaRE antibiotic categories among healthcare staff during the CwPAMS project. The means used to introduce the principles of WHO AWaRe included AMS train-the-trainer workshops; specific hospital meetings on the principles; AMS workshops; and Medicines and Therapeutic Committee (MTC) meetings.

### 3.4. Other AMS Activities

The respondents were asked to report any other actions related to AMS that were ongoing within their organisation. The following individual responses were received: Accreditation and implementation of the AMS training modules as CPD for healthcare workers; implementation of training, including those developed in the hospital and national training, drug audits and surveillance; implementation of the antibiogram; plans to engage hospital management and carry out the Global Point-Prevalence Survey (GPPS). Furthermore, also included was the formation of the Medicines Therapeutic Committee (MTC); the establishment of a community of practices; plans to resume implementation of the AMS strategy and work plan that has been on hold since the pandemic; and the development of guidelines and the publication of AMS manuscripts.

### 3.5. Barriers to AMS Implementation

The AMS checklist required the participants to select a maximum of six specified barriers to effective stewardship in their organisation. This also included an option for the participants to specify their own barrier if it was not listed. Table 3 shows the most important barriers selected by the participants. The same top five barriers were identified in both surveys from a list of 18 options. However, there was an increase in the proportion of respondents indicating a ‘lack of funding’ and ‘inadequate use of the microbiology laboratory’ as barriers post-intervention. In contrast, the proportion of respondents indicating ‘insufficient microbiology lab capacity’, ‘lack of motivated or engaged staff’, and ‘insufficient time for qualified personnel to perform stewardship’ as barriers decreased post-intervention. Two sites listed additional barriers that included: hierarchical barriers to pharmacists making interventions and a lack of resources.

### 3.6. Unique Contribution of Implementing the AMS Checklist

In the post-CwPAMS checklist, 10 sites provided further information in response to the open-ended questions, which indicated that the checklist had prompted them to take additional actions that were not part of the original AMS intervention plan of the CwPAMS project. The participants were invited to report up to five additional activities that they had engaged in based on the checklist. Across all sites, 29 additional AMS activities were listed. The examples included: the development of empirical guidelines; GPPS completion; GPPS training; the establishment of a multidisciplinary AMS team; a collection of baseline data on antimicrobial use; and the conduct of an AWaRe analysis of the antibiotic prescribing patterns at their hospital. The complete list is provided in Box 1.

Box 1 **Key interventions taken as a result of completing the pre-CwPAMS checklist (i.e., interventions not initially planned as part of the CwPAMS project).**

Cascading training to other health care providers in the hospital.

Institution of a Hospital Antimicrobial Stewardship Team.

Development of an adult empiric guideline based on common indications seen at the hospital. Annual guideline review.

Engaging with Community Durbars.

Global Point Prevalence Survey (GPPS).

Antibiotic Guidelines.

Radio Programmes November.

AMS Training Manual.

Surveillance

Auditing.

Dissemination of report.

GPPS Training

made a film focused on IPC and attendant behaviour.

Increase awareness of AMS.

Increase awareness of IPC

It enabled us to set out the goals of our partnership clearly.

To establish a multidisciplinary AMS team

To gather base line data on antimicrobial use

To conduct PPS training and studies

Conduct an AWARe analysis of antibiotic prescribing pattern at the hospital.

Conduct regular AMS training of the staff using the Scottish Triad approach

Establishing the MTC/

Plans to reconstitute alcohol hand rub.

Global Point Prevalence Study

Use of MicroGuide app to promote guidelines

Larger cohort of HPs trained.

Strengthening Hospital AMS Committee. Ensuring clarity on roles and responsibilities of AMS committee members.

Revising AMS committee membership, Developing a clear and timely action plan.

## 4. Discussion

Contextualised co-created AMS tools are necessary for managing the use of antimicrobials across different healthcare settings. Our work set out to develop and implement a new checklist of core AMS elements with a regional focus on Sub-Saharan Africa.

### 4.1. Development of the AMS Checklist

A modified Delphi process that included participants involved in partnerships of UK institutions with hospitals in Tanzania, Zambia, Uganda, and Ghana was used to ensure that the final AMS checklist was relevant and understandable for local healthcare staff in Sub-Saharan Africa. The final tool was cognizant of the unique settings in which they operate and the differences in practice from high-income settings. Compared to the original global checklist by Pulcini et al. [11], the AMS checklist developed in this study included a combination of closed-ended and open-ended questions that give room for a more comprehensive exploration of AMS activities.

The consensus process targeted the lower resource settings of LMICs in Sub-Saharan Africa by considering context-specific information and the involvement of experts from a broad range of specialities. Our work extends ongoing attempts to develop baseline assessment tools in Africa. This includes a Kenyan study in 2020, investigating the AMS policies and structures in 16 Kenyan hospitals while adapting the UK NICE AMS system to the Kenyan healthcare system [17]. Another study developed a survey questionnaire to investigate existing AMS activities for learners of the Massive Online Open Course (MOOC) [18,19].

By involving local healthcare staff in the development of our checklist, we also fostered a sense of ownership and commitment, thus serving as an example of successful co-creation. Compared to the LMIC AMS checklist contained within the WHO Practical Toolkit for healthcare facilities [12], which comprises 28 elements across six sections, our newly developed checklist contains 54 checklist items across eight main sections. While both checklists cover the more essential core elements for the National Antimicrobial Stewardship Programmes, including policy, guidelines and governance, awareness, education and training, IPC and surveillance, our newly developed tool was co-created and tested in the specific healthcare setting of Sub-Saharan Africa, thus increasing its acceptability amongst hospital staff and its level of contextualisation. Acceptability and contextualisation were prioritised to ensure the continued relevance of the tool and, consequently, the sustenance of antimicrobial stewardship activities in the long run. Similar tools are available in high-income countries, including the CDC Core Elements of Hospital Antibiotic Stewardship Programs developed in 2014 and updated in 2019 to reflect the new evidence from the field of antibiotic stewardship and the lessons learned from five years of experience [20]. The core elements in this checklist are similar to the developed checklist, each having sections on leadership, accountability, reporting, expertise, education, monitoring/tracking, and further actions. Additional checklist variations with adaptations to local healthcare settings could be developed following the modified Delphi consensus procedure employed in our study.

### 4.2. AMS Checklist Implementation

The initial results obtained from the use of our AMS checklist across 19 sites revealed large variations in the AMS’s capacities and local needs regarding support. Several healthcare sites had available expertise on infection management and stewardship, education and practical training, up-to-date recommendations for infection management, national antimicrobial prescribing guidelines, and continual monitoring and surveillance. Comparatively few sites, however, reported the presence of senior management leadership towards AMS, published AMS protocols, accountability structures, regular reporting and feedback, and routine ward rounds focused on infection and antimicrobial prescribing.

The results on the use of the checklist post-CwPAMS programme, including a range of interventions [9,21,22,23,24,25,26,27,28,29,30], indicate that the greatest improvements were observed in the core elements relating to senior AMS leadership, accountability, and responsibility. Smaller improvements were reported with regard to the availability of AMS expertise, education and practical training, monitoring and surveillance, and other AMS actions. The standout items included the availability of formal organisational multidisciplinary structures responsible for AMS and the conduct of point-prevalence surveys (79% and 71% improvements, respectively). At the other end of the spectrum, there were the availability of laboratory and imaging services and the presence of financial support for AMS activities, which only showed 7% and 14% improvements, respectively. The post-intervention checklist also demonstrated a better integration of pharmacists, nurses, and all clinical staff groups in AMS committees across the project sites. Local variations in improvements could be attributed to several factors, including a political and administrative will, workforce capacity and, importantly, funding.

The findings obtained through our contextualised AMS checklist mirror the published AMS reports in LMICs, which highlight the presence of national antimicrobial prescribing guidelines in the countries where the sites are located [21,31,32,33] but identify the challenges in AMR-specific education and training, diagnostic facilities, regulation of safety and efficacy of medications, and shortages of healthcare personnel and expertise [34,35,36,37]. Although these studies highlight the overall gaps in AMS implementation in LMICs, the differences observed across the regions, hospitals, and sites also demonstrate the need for case-by-case evaluations of AMS programmes for the development of appropriate and sustainable solutions. Our study suggests that the newly developed AMS checklist will enhance such evaluations across the wider region of Sub-Saharan Africa.

### 4.3. Barriers to and Opportunities for AMS Implementation

The initial findings from using the checklist across partnership sites reveal that the barriers that mostly existed pre-intervention were still major issues post-intervention. Some of these barriers (e.g., a lack of funding and insufficient microbiology lab capacity) could require major institutional changes to be overcome. However, other barriers (e.g., inadequate use of the microbiology laboratory and lack of motivated staff) highlight opportunities for further AMS intervention and workforce engagement.

In addition to identifying local AMS capacities and needs, our new checklist was successful in capturing the post-intervention changes in local programmes.

While some of the improvements noted above are the results of a funded AMS intervention that was part of the CwPAMS project, our end-of-project survey noted results that were not attributable to the initial CwPAMS project plans. Indeed, healthcare staff reported that the mere completion of our AMS checklist prompted them to engage in a revision of their AMS activities and led to important changes in their daily practice. Twenty-nine additional AMS activities were listed by the participating healthcare sites as having resulted from completing the checklist. While some of these activities (notably improved recommendations around infection control) may be explained by the global pressures of healthcare-associated infections and, latterly, the COVID-19 pandemic, other activities (e.g., the development of empirical guidelines around antibiotic prescribing) were directly related to AMS. Our results thus suggest that the newly developed contextualised AMS checklist has the potential to make a positive impact on the effectiveness of AMS interventions in Sub-Saharan Africa.

### 4.4. Strengths and Limitations

A key strength of this work was that the study employed a modified Delphi consensus process, which is a standard method of developing checklists or similar tools and has been widely used for designing AMS programmes in hospitals. We engaged local stakeholders, including senior management, frontline healthcare professionals, and public health specialists, in the consensus process, thus increasing elements of co-creation and a subsequent sense of ownership for the materials. This also meant that the checklist modifications were context-specific and relevant to health institutions. The representatives were recruited based on their level of expertise, level of interest, heterogeneity, accessibility, and the intended size of the panel, as recommended by the existing literature [38,39]. Furthermore, our selection strongly considered representativeness as it engaged stakeholders across all partnership sites in all countries.

This study, which commenced prior to the publication of the WHO AMS toolkit for LMICs, provides an additional step toward increasing the suitability and acceptance of standardised AMS tools in LMIC settings in several different ways:

(1) Our narrow focus on Sub-Saharan Africa (specifically Tanzania, Zambia, Uganda, and Ghana) allows for further local adaptation of the materials.
(2) Our unique methodological approach uses elements of co-creation through the strong involvement of local hospital representatives during a Delphi consensus procedure.
(3) We extended the number of checklist items to capture the more nuanced differences in the AMS elements. Additionally, we incorporate open-ended questions within the AMS checklist to allow for more reporting flexibility.
(4) We provide an initial evaluation of the checklist’s effectiveness by using it to measure the outcomes of an AMS intervention programme.

The Delphi process was limited by not having an opportunity for face-to-face discussions about specific items. The small number of study sites could be considered a limitation; however, the similarity of the results across multiple countries suggests the results are transferable and that the approach can be implemented within other countries.

## 5. Conclusions

Our study has tested a successful methodology for making regional adaptations to global AMS tools and demonstrated the effectiveness of a contextualised AMS checklist in the challenging healthcare setting of Sub-Saharan Africa. This effectiveness was shown to go beyond the mere capture of the AMS changes following an intervention. Indeed, our results suggested that completing the checklist prompted local healthcare providers to review their initiatives and increase their AMS efforts. Our AMS checklist is widely available for use in health partnerships and institutions and extends the existing tools, such as Pulcini et al.’s [11] global AMS checklist and the WHO LMIC toolkit [24].

## Figures and Tables

**Table 1:** Comparison of selected AMS checklist results pre- and post-AMS intervention that formed part of the CwPAMS projects. The numbers indicate the total number of sites that agreed with the item in question. Percentages (out of 14 sites) are provided alongside the numbers. The final column shows post-intervention improvement through the percentage increase.

	Pre-AMS Intervention *N* = 14		Post-AMS Intervention *N* = 14		Percentage Difference
Has your hospital management formally identified AMS as a priority objective for the institution and included it in its key performance indicators?	**2**	14%	10	71%	+57%
Is there dedicated, sustainable and sufficient budgeted financial support for AMS activities (e.g., support for salary, training, or IT (information technology) support)?	1	7%	3	21%	+14%
Does your hospital have a formal organisational multidisciplinary structure responsible for AMS?	3	21%	14	100%	+79%
Does your hospital have a dedicated committee focussed on antimicrobial use?	2	14%	8	57%	+43%
Is there a healthcare professional identified as a leader for AMS activities at your hospital and responsible for implementing the programme?	4	29%	12	86%	+57%
Is a multidisciplinary AMS team available at your hospital (e.g., greater than one trained staff member supporting clinical decisions to ensure appropriate antimicrobial use) to implement your stewardship strategy?	1	7%	10	71%	+64%
Are clinicians, nurses or pharmacists, other than those part of the AMS team (e.g., from the ICU, Internal Medicine and Surgery) involved in the AMS committee?	1	7%	9	64%	+57%
Do you have access to laboratory/imaging services to be able to support the diagnosis of the most common infections at your hospital?	8	57%	9	64%	+7%
Are the results available in a timely manner to be able to support diagnosis of most common infections?	3	21%	6	43%	+22%
In your hospital are there, or do you have access to healthcare professionals in infection management and stewardship willing to constitute an antimicrobial stewardship team?	9	64%	12	86%	+22%
Does your hospital offer access to educational resources to support staff training on how to optimise antimicrobial prescribing?	2	14%	6	43%	+29%
Does your hospital monitor the quantity of antimicrobials prescribed/dispensed/purchased at the unit and/or hospital wide level?	5	36%	9	64%	+28%
Does your stewardship programme monitor compliance with one or more of the specific interventions put in place by the stewardship team (e.g., indication captured in the medical record for all antimicrobial prescriptions, or antibiotic prescribed follows hospital guidelines)?	1	7%	7	50%	+43%
Has your hospital conducted a point prevalence survey (PPS) for antimicrobial use in the last year?	1	7%	11	79%	+72%
Are hospital-specific reports on the quantity of antimicrobials prescribed/dispensed/purchased shared with/fed back to prescribers?	3	21%	7	50%	+29%
Does your stewardship programme share facility-specific reports on antibiotic susceptibility rates with prescribers?	3	21%	5	36%	+15%
Are results of audits/reviews of the quality/appropriateness of antimicrobial use communicated directly with prescribers?	1	7%	7	50%	+43%
Does your hospital have available and up-to-date recommendations for infection management (diagnosis, prevention and treatment)?	7	50%	10	71%	+21%
Do you have any published AMS protocols e.g., restricted antimicrobial list, IV to oral policy (that have been ratified for use within your organisation)?	0	0%	5	36%	+36%
Do you have any published Infection Prevention and Control protocols e.g., hand hygiene, WASH (that have been ratified for use in your health institution)?	7	50%	12	86%	+36%
Are there regular infection and antimicrobial prescribing focused ward rounds in specific departments in your hospital?	0	0%	3	21%	+21%
Does the organisation have local/hospital specific antimicrobial prescribing guidelines? This may be included as part of a wider drug formulary.	3	21%	7	50%	+29%

**Table 2:** Number of members by profession of multidisciplinary AMS teams, pre- and post-AMS intervention at CwPAMS project sites.

AMS Team Members	Pre-AMS Intervention	Post-AMS Intervention	Total Increase Post-Intervention
Pharmacists	3	13	10
Nurses	3	14	11
Clinicians	3	11	8
Infectious Disease doctors	2	6	4
Surgeons	0	6	6
Clinical microbiologists	1	2	1
Laboratory microbiologists	0	9	9
ITU consultants	0	0	0
Data analysts	1	3	2
Infection control staff	2	7	5

**Table 3:** Top five barriers to AMS pre- and post-AMS intervention.

Priority	Top 5 Barriers to AMS Selected by Participants Pre- and Post-CwPAMS Intervention (Pre-AMS Intervention)
1	Lack of funding
2	Insufficient microbiology lab capacity
3	Qualified personnel do not have enough time to perform stewardship
4	Inadequate use of the microbiology laboratory
5	Lack of motivated or engaged staff

## Data Availability

Not applicable.

## Supplementary Materials

The following supporting information can be downloaded at: https://www.mdpi.com/article/10.3390/healthcare10091706/s1. Supplementary Material S1: Consensus procedure; Supplementary Material S2: Differences in the AMS Checklist compared to the checklist by Pulcini et al.; Supplementary Material S3: Hospital sites where the research was conducted; Supplementary Material S4: Baseline AMS checklist data from non-CwPAMS sites.
