# Supplementary material for: Development and Implementation of an Antimicrobial Stewardship Checklist in Sub-Saharan Africa: A Co-Creation Consensus Approach
Source: Healthcare (Basel). 2022 Sep 6;10(9):1706. doi: 10.3390/healthcare10091706 (PMC9498699; doi:10.3390/healthcare10091706)
Supplement: Supplementary file 1 [file healthcare-10-01706-s001.zip › healthcare-1853935-supplementary.pdf]

Supplementary materials

S1: Supplementary material 1: Consensus procedure

In February 2019, the 14 representatives were invited to complete an online survey based on core indicators and core elements of ASPs, developed from the published international consensus [11]. The form contained 10 main sections of questions or items with the first section containing questions on core indicators of ASPs specific to CwPAMS. The next seven sections included questions under the 7 core elements of hospital ASPs with the same headings as in the core elements developed by Pulcini et al [12]. This was followed by a section of concluding questions and a final section on behaviours for AMS (that was used to prepare another tool specific to behaviours for AMS; reported in another paper). Participants were invited to score each question in each section on a scale of 1 to 5 with 1 signifying 'not at all important' and 5 signifying 'completely important'. Participants were also given an option to select an 'I do not understand this indicator' (IDU) response. In this way the participants scored how important or not each question was to be included in the final checklist as part of the CwPAMS project. At the end of each set of questions in each section, participants were allowed to provide any further comments on the questions, which could be suggestions, clarifications or any other issues they felt were important to raise about the question. Finally, there was another textbox in each section for participants to suggest any questions that they felt should be added to the checklist.

Items on the AMS checklist (sections 1 to 9) were selected if participants gave a question or item a score of ≥4. Behaviours in the last section of the checklist were used to prepare a behaviour tool if they were chosen by ≥ 10 respondents. After this first round of delivering the survey to the participants, a new format of the AMS checklist was created that took into consideration the participants' comments and suggestions for rephrasing, inclusion and exclusion of questions. The new checklist was presented as a survey developed with Survey Monkey and was shared with one of the members of the partnerships and AMS experts to gather suggestions for rephrasing, or additional changes in elements of the checklist. This led to the development of a final AMS checklist tool.

Development of the tool

12 representatives of the CwPAMS partnerships responded to the online survey in early March 2019; 2 representatives responded in late March, but their responses and opinions were taken into consideration in the final survey development.

The first survey of the AMS checklist contained 9 main sections (not including introductory questions on the partnerships) , including a section on 'core indicators' for the CwPAMS project, followed by the seven sections on the core elements of hospital ASPs, and then a last section of 'concluding questions'. A total of 53 individual questions/checklist items were composed in the 9 main sections, with comments boxes at the end of each section included separately. Following the initial round of the survey responses, all questions in the 9 sections of the survey were selected (i.e. they were given an average score ≥4) by the 14 representatives. Overall comments were made by the representatives on the repetition of questions in the first section (CwPAMS core indicators) with those in the rest of the survey. Hence, it was suggested that the questions in the first section be merged into other sections as applicable to avoid this repetition. Suggestions were made for rephrasing some of the questions and the inclusion of 24 new questions in the checklist.

The original checklist by Pulcini et al 12 contains the 7 core elements of hospital AMS programmes, with a total of 29 checklist items spread across all the core elements. Following the consensus process, 6 of the checklist items from the original checklist were removed, and a further 8 checklist items were rephrased for clarification. 25 additional checklist items were added based on the suggestions and comments by the participants.

Following the removal of the core indicators section with the embedding of the questions in this section into other sections, and piloting of the tool, the final AMS checklist tool contained 54 items across 8 main sections (7 sections on the core elements of hospital AMS programmes and the concluding section).

Table S1 is an outline of the number of items in each section (sections 1 to 9) which were available for scoring by participants in the first survey and the number of questions in the second survey (with a short description of the changes made).

Table S1: **Sections of the first and second AMS Checklist surveys**

Section title	Number of questions in first survey	Number of questions in second survey	Changes made
1: Core Indicators (CwPAMS)	15	0	Section removed, and questions embedded into other sections
2: Senior management leadership towards AMS	3	3	All questions retained
3: Accountability and responsibilities	8	13	5 questions added
4: Available expertise on infection management and stewardship	2	3	1 question added (the first question in the first survey was divided into two separate questions)
5: Education and practical training	4	11	8 questions added; 1 question removed
6: Continual monitoring and surveillance	5	5	1 question was added; 1 question was removed
7: Regular reporting and feedback	3	3	All q uestions retained
8: Other actions aiming at responsible antimicrobial use	8	10	8 questions added; 6 questions removed

9: Concluding questions of checklist	5	6	1 question added

S2: Supplementary material 2: Differences in the AMS Checklist compared to the checklist by Pulcini et al[12]

Added checklist items- **Green-25**

Deleted checklist items- **Red-6**

Rephrased checklist items- **Amber- 8**

Pulcini et al [12]	AMS checklist tool
1. Senior hospital management leadership towards antimicrobial stewardship<br>1.1. Has your hospital management formally identified antimicrobial stewardship as a priority objective for the institution and included it in its key performance indicators?<br>1.2. Is there dedicated, sustainable and sufficient budgeted financial support for antimicrobial stewardship activities (e.g., support for salary, training, or IT (information technology) support)?<br>1.3. Does your hospital follow any (national or international) staffing standards for antimicrobial stewardship activities (e.g. number of full-time equivalent (FTE) per 100 beds for the different members of the antimicrobial stewardship team)?	1. Senior hospital management leadership towards antimicrobial stewardship<br>1.1.<br>Has your hospital management formally identified AMS as a priority objective for the institution and included it in its key performance indicators?<br>1.2.<br>Is there dedicated, sustainable and sufficient budgeted financial support for AMS activities (e.g., support for salary, training, or IT (information technology) support)?<br>1.3.<br>Does your hospital follow any (national or international) staffing standards for AMS activities (e.g. number of full-time equivalent (FTE) per 100 beds for the different members of the AMS team)?
2. Accountability and responsibilities<br>2.1.<br>Does your hospital have a formal/written antimicrobial stewardship programme/strategy accountable for ensuring appropriate antimicrobial use?	2. Accountability and responsibilities<br>2.1.<br>Does your hospital have a formal/written AMS programme/strategy accountable for ensuring appropriate antimicrobial use?

2.2.<br><br>Does your hospital have a formal organizational multidisciplinary structure responsible for antimicrobial stewardship (e.g., a committee focused on appropriate antimicrobial use, pharmacy committee, patient safety committee or other relevant structure)?<br><br>2.3.<br><br>Is there a healthcare professional identified as a leader for antimicrobial stewardship activities at your hospital and responsible for implementing the programme?<br><br>2.4.<br><br>Is there a document clearly defining roles, procedures of collaboration and responsibilities of the antimicrobial stewardship team members?<br><br>2.5.<br><br>Are clinicians, other than those part of the antimicrobial stewardship team (e.g. from the ICU, Internal Medicine and Surgery) involved in the antimicrobial stewardship committee?<br><br>2.6.<br><br>Does the antimicrobial stewardship committee produce regularly (indicate minimum time) a dedicated report which includes e.g. antimicrobial use data and/or prescription improvement initiatives, with time-committed short term and long term measurable goals/targets for optimising antimicrobial use?<br><br>2.7.<br><br>Is there a document clearly defining the procedures of collaboration of the antimicrobial stewardship team/committee with the infection	2.2.<br><br>Does your hospital have a formal organisational multidisciplinary structure responsible for AMS (e.g., a committee that focuses on OR takes responsibility for appropriate antimicrobial use, pharmacy committee, patient safety committee, infection prevention and control committee or other relevant structure)?<br><br>2.3.<br><br>If yes, what is the structure?<br><br>2.4.<br><br>Is there a healthcare professional identified as a leader for AMS activities at your hospital and responsible for implementing the programme?<br><br>2.5.<br><br>Please provide more details about the AMS leader e.g. main role, how much time is available to dedicate to AMS activities etc.<br><br>2.6.<br><br>Is a multidisciplinary AMS team available at your hospital (e.g., greater than one trained staff member supporting clinical decisions to ensure appropriate antimicrobial use) to implement your stewardship strategy?<br><br>2.7.<br><br>Is there a document clearly defining roles, procedures of collaboration and responsibilities of the AMS team members?<br><br>2.8.<br><br>Are clinicians, nurses or pharmacists, other than those part of the AMS team (e.g. from the ICU, Internal Medicine and Surgery) involved in the AMS committee?

prevention and control team/committee?	2.9.<br><br>Does the AMS committee produce a regular (e.g. every 6 months) dedicated report which includes e.g. antimicrobial use data and/or prescription improvement initiatives, with time-committed short term and long-term measurable goals/targets for optimising antimicrobial use?<br><br>2.10.<br><br>Is there a document clearly defining the procedures of collaboration of the AMS team/committee with the infection prevention and control team/committee?<br><br>2.11.<br><br>Who are the members of the AMS team?<br><br>2.12.<br><br>If there is a committee responsible for AMS, how often does the committee meet?<br><br>2.13.<br><br>Are minutes or notes taken during the meetings?
3. Available expertise on infection management<br><br>3.1.<br><br>Do you have access to laboratory/imaging services and to timely results to be able to support the diagnosis of the most common infections at your hospital?<br><br>3.2.<br><br>In your hospital are there, or do you have access to, trained and experienced healthcare professionals (medical doctor, pharmacist, nurse …) in infection management (diagnosis, prevention and treatment) and stewardship willing to constitute an antimicrobial stewardship team?	3. Available expertise on infection management<br><br>3.1.<br><br>Do you have access to laboratory/imaging services to be able to support the diagnosis of the most common infections at your hospital?<br><br>3.2.<br><br>Are the results available in a timely manner to be able to support diagnosis of most common infections?<br><br>3.3.<br><br>In your hospital are there, or do you have access to healthcare professionals (medical doctor, pharmacist, nurse etc.)

	in infection management (diagnosis, prevention and treatment) and stewardship willing to constitute an antimicrobial stewardship team?
4. Education and practical training<br><br>4.1.<br><br>Does your hospital offer a range of educational resources to support staff training on how to optimize antimicrobial prescribing?<br><br>4.2.<br><br>Do the antimicrobial stewardship team members receive regular training in antimicrobial prescribing and stewardship?	4. Education and practical training<br><br>4.1.<br><br>Does your Ministry of Health/Department of Health offer any educational resources to support staff training on how to optimise antimicrobial prescribing?<br><br>4.2.<br><br>Does your hospital offer access to educational resources to support staff training on how to optimise antimicrobial prescribing?<br><br>4.3.<br><br>Do the AMS team members receive regular training in antimicrobial prescribing and stewardship?<br><br>4.4.<br><br>Does your hospital have induction for clinical staff?<br><br>4.5.<br><br>If yes, does this training include IPC and/or AMS and resistance training?<br><br>4.6.<br><br>If yes, is this training mandatory?<br><br>4.7.<br><br>How often is the training on antibiotic use and stewardship conducted or made available to staff? (Please estimate the frequency in times per month or year)<br><br>4.8.<br><br>How is the training delivered? (Select all that apply)<br><br>4.9.

	In the last year, who have been the provider of training on antimicrobial stewardship? E.g. in country organisations, International organisations/charities/professional bodies? Please provide information including names and how training was delivered (where applicable)<br><br>4.10.<br><br>What was the total number of each clinical staff trained in the last year<br><br>4.11.<br><br>What is the estimated total number of each clinical staff in the hospital
5. Other actions aiming at responsible antimicrobial use<br><br>5.1.<br><br>Is a multidisciplinary antimicrobial stewardship team available at your hospital (e.g., greater than one trained staff member supporting clinical decisions to ensure appropriate antimicrobial use)?<br><br>5.2.<br><br>Does your hospital support the antimicrobial stewardship activities/strategy with adequate information technology services?<br><br>5.3.<br><br>Does your hospital have an antimicrobial formulary (i.e. a list of antimicrobials that have been approved for use in a hospital, specifying whether the drugs are unrestricted, restricted (approval of an antimicrobial stewardship team member is required) or permitted for specific conditions)?<br><br>5.4.<br><br>Does your hospital have available and up-	5. Other actions aiming at responsible antimicrobial use<br><br>5.1.<br><br>Does your hospital have available and up-to-date recommendations for infection management (diagnosis, prevention and treatment)?<br><br>5.2.<br><br>Do you have any published AMS protocols e.g. restricted antimicrobial list, IV to oral policy (that have been ratified for use within your organisation)?<br><br>5.3.<br><br>Do you have any published Infection Prevention and Control protocols e.g. hand hygiene, WASH (that have been ratified for use in your health institution)?<br><br>5.4.<br><br>Are there regular infection and antimicrobial prescribing focused ward rounds in specific departments in your hospital?<br><br>5.5.<br><br>Does the organisation have local/hospital

to-date recommendations for infection management (diagnosis, prevention and treatment), based on international/national evidence-based guidelines and local susceptibility (when possible), to assist with antimicrobial selection (indication, agent, dose, route, duration) for common clinical conditions?<br><br>5.5.<br><br>Does your hospital have a written policy that requires prescribers to document an antimicrobial plan (includes indication, name, dosage, duration, route and interval of administration) in the medical record or during order entry for all antimicrobial prescriptions?<br><br>5.6.<br><br>Does the antimicrobial stewardship team review/audit courses of therapy for specified antimicrobial agents or clinical conditions at your hospital?<br><br>5.7.<br><br>Is advice from antimicrobial stewardship team members easily available to prescribers?<br><br>5.8.<br><br>Are there regular infection and antimicrobial prescribing focused ward rounds in specific departments in your hospital?	specific antimicrobial prescribing guidelines? This may be included as part of a wider drug formulary.<br><br>5.6.<br><br>If yes, do these guidelines include WHO AWaRe* categories? *WHO AWaRe antibiotics are antibiotics in the 'Access', 'Watch' and 'Reserve' groups of antibiotics in the WHO essential medicines list. 'Access' antibiotics are first and second line antibiotics for common infections 'Watch' antibiotics are antibiotics indicated for only specific infections as they have a greater risk of bacterial resistance 'Reserve' antibiotics are last-resort antibiotics such as colistin ESCMID study group for antimicrobial stewardship, 2017 http://esgap.escmid.org/? p=1550<br><br>5.7.<br><br>Has the organisation formally adopted the use of national antimicrobial prescribing guidelines (where available)<br><br>5.8.<br><br>Please list the antimicrobial prescribing or stewardship guidelines/tools/protocols you currently have /use in your organisation. Please state exact names of documents, including organisation name and date of publication/review (if available). If none - please state none.<br><br>E.g. IV to Oral Switch Policy - xxxx hospital - date of review January 2018<br><br>E.g. how to use third line antibiotics, guidelines on appropriate use of IV antibiotics, written procurement process to avoid stockouts, clinical guidelines on AMR, guidelines on prescribing

	of antimicrobials, surgical prophylaxis guidelines.<br>5.9.<br>Please list the infection prevention and control protocols you currently have in your organisation. Please state exact names of documents, including organisation name and date of publication/review (if available). If none - please state none.<br>E.g. Hand Hygiene policy - xxxx hospital - date of review January 2018<br>5.10.<br>How many guidelines/tools are currently available for infection management or antimicrobial prescribing/stewardship?
6. Continual monitoring and surveillance<br>6.1.<br>Does your hospital monitor the quality of antimicrobial use at the unit and/or hospital wide level?<br>6.2.<br>Does your stewardship programme monitor compliance with one or more of the specific interventions put in place by the stewardship team (e.g. indication captured in the medical record for all antimicrobial prescriptions)?<br>6.3.<br>Does your hospital monitor antibiotic susceptibility rates for a range of key bacteria?<br>6.4.<br>Does your hospital monitor the quantity of antimicrobials prescribed/dispensed/purchased at the unit and/or hospital wide level?	6. Continual monitoring and surveillance<br>6.1.<br>Does your hospital monitor the quantity of antimicrobials prescribed/ dispensed/purchased at the unit and/or hospital wide level?<br>6.2.<br>If yes, how is this done?<br>6.3.<br>Does your stewardship programme monitor compliance with one or more of the specific interventions put in place by the stewardship team (e.g. indication captured in the medical record for all antimicrobial prescriptions, or antibiotic prescribed follows hospital guidelines)?<br>6.4.<br>Does your hospital monitor antibiotic susceptibility rates for a range of key bacteria?

	6.5.<br><br>Has your hospital conducted a point prevalence survey (PPS) for antimicrobial use in the last year?
7. Reporting and feedback (on a continuous basis)<br><br>7.1.<br><br>Does your stewardship programme share hospital-specific reports on the quantity of antimicrobials prescribed/dispensed/purchased with prescribers?<br><br>7.2.<br><br>Does your stewardship programme share facility-specific reports on antibiotic susceptibility rates with prescribers?<br><br>7.3.<br><br>Are results of audits/reviews of the quality/appropriateness of antimicrobial use communicated directly with prescribers?	7. Reporting and feedback (on a continuous basis)<br><br>7.1.<br><br>Are hospital-specific reports on the quantity of antimicrobials prescribed/dispensed/purchased shared with/fedback to prescribers?<br><br>7.2.<br><br>Does your stewardship programme share facility specific reports on antibiotic susceptibility rates with prescribers?<br><br>7.3.<br><br>Are results of audits/reviews of the quality/appropriateness of antimicrobial use communicated directly with prescribers?

S3: Supplementary material 3: Hospital sites where the research was conducted

The names of the sites are as follows:

(1) Fort Portal Regional Referral Hospital (FPRRH);

(2) Lekma Hospital;

(3) Assemblies of God Hospital, Saboba;

(4) Korle Bu Teaching Hospital;

(5) Ho Teaching Hospital, Ghana;

(6) Jinja Regional Referral Hospital;

(7) KCMC Tanzania;

(8) Mulago specialized Women and neonatal hospital and Kawempe Hospital (Kampala);

(9) Keta Municipal Hospital;

(10) Ghana Police Hospital;

(11) St Mary's Hospital Lacor;

(12) Gulu Regional Referral Hospital;

(13) Entebbe Hospital;

(14) University teaching hospitals ,Lusaka;

(15) Mbarara regional referral hospital, western Uganda;

(16) Kabale Regional Referral Hosptial, Western Uganda;

(17) Mubende RRH, Mubende District;

(18) Arua RRH;

(19) Mbale RRH in Eastern Uganda.

S4: Supplementary material 4: Baseline AMS checklist data from non CwPAMS sites

	**Yes**<br>**N=5**
Has your hospital management formally identified AMS as a priority objective for the institution and included it in its key performance indicators?	4 (80%)
Is there dedicated, sustainable and sufficient budgeted financial support for AMS activities (e.g., support for salary, training, or IT (information technology) support)?	0
Does your hospital have a formal organisational multidisciplinary structure responsible for AMS?*	5 (100%)
Is there a healthcare professional identified as a leader for AMS activities at your hospital and responsible for implementing the programme?	3 (60%)
Is a multidisciplinary AMS team available at your hospital (e.g., greater than one trained staff member supporting clinical decisions to ensure appropriate antimicrobial use) to implement your stewardship strategy?	2 (40%)
Are clinicians, nurses or pharmacists, other than those part of the AMS team (e.g. from the ICU, Internal Medicine and Surgery) involved in the AMS committee?	3 (60%)
Do you have access to laboratory/imaging services to be able to support the diagnosis of the most common infections at your hospital?	5 (100%)
Are the results available in a timely manner to be able to support diagnosis of most common infections?	0
In your hospital are there, or do you have access to healthcare professionals in infection management and stewardship willing to constitute an antimicrobial stewardship team?	5 (100%)
Does your hospital offer access to educational resources to support staff training on how to optimise antimicrobial prescribing?	2 (40%)
Does your hospital monitor the quantity of antimicrobials prescribed/ dispensed/purchased at the unit and/or hospital wide level?	2 (40%)
Does your stewardship programme monitor compliance with one or more of the specific interventions put in place by the stewardship team (e.g. indication captured in the medical record for all antimicrobial prescriptions, or antibiotic prescribed follows hospital guidelines)?	2 (40%)

Has your hospital conducted a point prevalence survey (PPS) for antimicrobial use in the last year?	4 (80%)
Are hospital-specific reports on the quantity of antimicrobials prescribed/dispensed/purchased shared with/ fed back to prescribers?	4 (80%)
Does your stewardship programme share facility-specific reports on antibiotic susceptibility rates with prescribers?	3 (60%)
Are results of audits/reviews of the quality/appropriateness of antimicrobial use communicated directly with prescribers?	2 (40%)
Does your hospital have available and up-to-date recommendations for infection management (diagnosis, prevention and treatment)?	2 (40%)
Do you have any published AMS protocols e.g. restricted antimicrobial list, IV to oral policy (that have been ratified for use within your organisation)?	0
Do you have any published Infection Prevention and Control protocols e.g. hand hygiene, WASH (that have been ratified for use in your health institution)?	5 (100%)
Are there regular infection and antimicrobial prescribing focused ward rounds in specific departments in your hospital?	2 (40%)
Does the organisation have local/hospital specific antimicrobial prescribing guidelines? This may be included as part of a wider drug formulary.	2 (40%)

* All hospitals reported having a Medicines and Therapeutics Committee (MTC) which oversees antimicrobial use, two hospitals reported that there was a dedicated committee focussed on antimicrobial use and one hospital reported there was an infection prevention and control committee which oversees antimicrobial use.
